# Outcomes of the management of synchronous rectal and prostate cancer: a systematic review

**DOI:** 10.1007/s00384-025-04992-w

**Published:** 2025-09-11

**Authors:** Ahmed Nassar, Noha E. Aly, Mootaz Elhusseini, Craig Parnaby, Emad Aly

**Affiliations:** 1 University of Aberdeen, Aberdeen, AB24 2ZD Scotland UK; 2 Department of General Surgery, Aberdeen Royal Infirmary, Aberdeen, Scotland UK; 3 Department of General Surgery, Poole Hospital, Poole, England UK; 4 Ain Shams University, Cairo, Egypt

**Keywords:** Synchronous rectal and prostate cancer, Coexisting rectal and prostate cancer, Rectal and prostate cancer

## Abstract

**Background:**

The optimal management of synchronous rectal cancer (RC) and prostate cancer (PC) remains unclear. This systematic review evaluates treatment strategies and reports postoperative, oncological, and quality-of-life outcomes in patients treated with curative intent.

**Methods:**

Following PRISMA guidelines, this systematic review was registered in PROSPERO (CRD42024598049). A search of Ovid MEDLINE, Embase, CENTRAL, and CDSR (inception to February 2025) identified randomised controlled trials and observational studies on synchronous RC and PC. Synchronous disease was defined as diagnosis or treatment initiation within 12 months. Patients with incurable RC were excluded. Treatment strategies and surgical approaches were analysed, with postoperative, oncological, and survival outcomes assessed.

**Results:**

Eight retrospective studies (124 patients) were included. Common treatments included pelvic chemoradiotherapy (CRT) followed by surgery (29%), prostate booster radiotherapy with CRT (24.2%), prostate brachytherapy (8%), and no prostate-directed treatment (11.3%). Surgical approaches included total mesorectal excision (TME) (74.4%), TME with prostatectomy (10.8%), and en-bloc pelvic exenteration (8%). Primary anastomosis was achieved in 61.8%, with 70% requiring a diverting stoma. Anastomotic leaks occurred in 10.8%, and severe complications (grades 3b–5) affected 15.4%, with fewer in robotic-assisted surgery (8.3%). R0 resection was achieved in 92.8%, with no difference between robotic and non-robotic groups. Local recurrence and distant metastasis occurred in 5.9% and 27%, respectively.

**Conclusion:**

There is no consistent approach for managing synchronous RC and PC. High-dose prostate radiotherapy may not improve survival and may increase postoperative complications. Robotic-assisted resections may reduce major complications without compromising oncological outcomes.

**Supplementary Information:**

The online version contains supplementary material available at 10.1007/s00384-025-04992-w.

## Introduction

Prostate and colorectal cancers are amongst the most common malignancies affecting males. In the UK alone, approximately 7,515 men are diagnosed with rectal cancer (RC) and 55,093 with prostate cancer (PC) annually [1]. It has been reported that synchronous RC occurs in 1.7 out of every 1,000 males diagnosed with PC which is a considerable number given the rising global incidence of both cancers [2, 3]. The prevalence of these cases is expected to rise due to several factors, including wider adoption of screening programs for both diseases, increased life expectancy, and the growing use of pelvic MRI for local staging of RC [2]. Among men newly diagnosed with PC who underwent screening colonoscopy, approximately 3% were also diagnosed with colorectal cancer [4]. Since RC accounts for about 30% of colorectal cancer cases [5], this suggests that 1 in every 100 patients with PC will also have synchronous RC. This figure has been supported by a recent study showing an upward trend of synchronous disease since 2011 [6].

Rectal and prostate cancers are distinct in their biology, behaviour, treatment strategies, and prognosis. Treatment planning becomes particularly challenging when advanced PC necessitates radiotherapy or surgical resection in the context of co-existing RC, which may require neoadjuvant chemoradiotherapy (CRT) followed by surgical resection or immediate surgical resection without prior CRT. Radiotherapy (RT) dosing and target organ planning differ significantly between RC and PC. Prostate cancer requires higher doses of RT, which can complicate RC resections and increase the risk of complications, including issues with rectal and urological anastomoses [7].

There is no consensus on the optimal surgical approach for managing synchronous RC and PC [8]. It is unclear whether these cases are best treated with pelvic exenteration involving en bloc resection, separate synchronous resections, or TME only with or without radical prostatic radiotherapy [9].

Given the limited available evidence on the optimal management of synchronous RC and PC, this review aims to evaluate the various treatment strategies that are currently used and report on post-operative, oncological and quality of life outcomes for management of synchronous RC and PC with curative intent.

## Methods

### Search strategy and selection criteria

This systematic review adhered to the Preferred Reporting Items for Systematic Reviews and Meta-Analyses (PRISMA) guidelines [10] and was registered in PROSPERO (registration no. CRD42024598049). The search included English-language studies published from the inception of literature (1946) to February 2025. Eligible studies consisted of randomised controlled trials (RCTs) and observational studies evaluating outcomes of different treatment approaches for patients diagnosed with synchronous RC and PC. Only studies involving patients with RC managed with curative intent were included. Studies focusing exclusively on non-synchronous cancers, colonic cancers requiring resections other than TME, or metastatic RC unsuitable for curative resection were excluded.

The search was conducted on August 8, 2024, by a senior information specialist from the Royal College of Surgeons of England. Database searches were performed in Ovid MEDLINE, Embase, the Cochrane Central Register of Controlled Trials (CENTRAL), and the Cochrane Database of Systematic Reviews (CDSR) using the Patient, Intervention, Comparison, Outcome (PICO) framework. To identify any newly published relevant studies, the search was updated on February 19, 2025, using the same criteria and conducted by the same team. Supplementary Table 1 outlines the PICO framework, while Supplementary Table 2 details the complete electronic search strategy.

Two independent reviewers (ME and NEA) conducted the abstract screening, with conflicts resolved by a third reviewer (AN). The same process was followed for the full-text review. Data collection was performed by two reviewers, and the results were verified by a third reviewer.

### Data analysis

Synchronous presentation was defined as the diagnosis of both cancers simultaneously or at the initiation of primary treatment for one cancer within 12 months of the other's diagnosis. This broader timeframe was chosen to reflect real-world clinical variability, including delays in diagnosis, staged investigations, referral processes, and multidisciplinary planning, where the management of both cancers is highly likely to overlap. The collected data included study details, patient demographics, disease-specific information for RC and PC, treatment details, and outcomes. Important definitions used in this study were summarised in supplementary Table 3.

The study aimed to evaluate three main categories of outcomes: 1) 30-day postoperative outcomes, 2) oncological outcomes, and 3) long-term quality-of-life outcomes. Postoperative outcomes included the risk of anastomotic leak, the need for a permanent stoma, length of hospital stay, and the occurrence of postoperative complications classified using the Clavien-Dindo system [11]. Oncological outcomes assessed the achievement of R0 resection, locoregional and distant recurrence for RC and PC, cancer-specific mortality (RC-related and PC-related), 3-year disease-free survival (DFS) for both cancers, and overall survival (OS). Quality-of-life outcomes focused on long-term complications impacting patients'well-being, such as urethral strictures and urinary incontinence. Sexual dysfunction was not reported as it was not assessed in all the studies included in this review. Prostate-related symptoms were evaluated using classification systems like the International Prostate Symptom Score (I-PSS) [12] and the Common Terminology Criteria for Adverse Events (CTCAE) grading system used for RC and PC [13].

Where possible, these outcomes were compared based on treatment strategies, types and doses of CRT, extent and types of surgical resections, and surgical approaches (open, laparoscopic or robotic assisted).

Observational studies were evaluated using the Mathes and Pieper criteria [14] and the Joanna Briggs Institute (JBI) appraisal tool for risk of bias assessment [15]. The overall risk of bias for each study was determined based on the number of questions answered as"yes,""no,"or"unclear."Studies were categorised as having a low risk of bias if no questions were answered unfavourably, moderate concern if one to two questions were answered unfavourably, and high risk if three or more questions were answered unfavourably.

### Statistical analysis

Non-continuous variables were summarized using counts, percentages, and ratios, while continuous data were represented as medians with interquartile ranges (IQR) as reported in individual studies. When aggregating data across studies, the median and the range of median values were used to summarize continuous variables. Chi-Square test was performed to compare categorical data within the study. A P value of less that 0.05 was considered of statistical significance.

Role of funding sources: There was no funding source for this study.

## Results

**Fig. 1:**
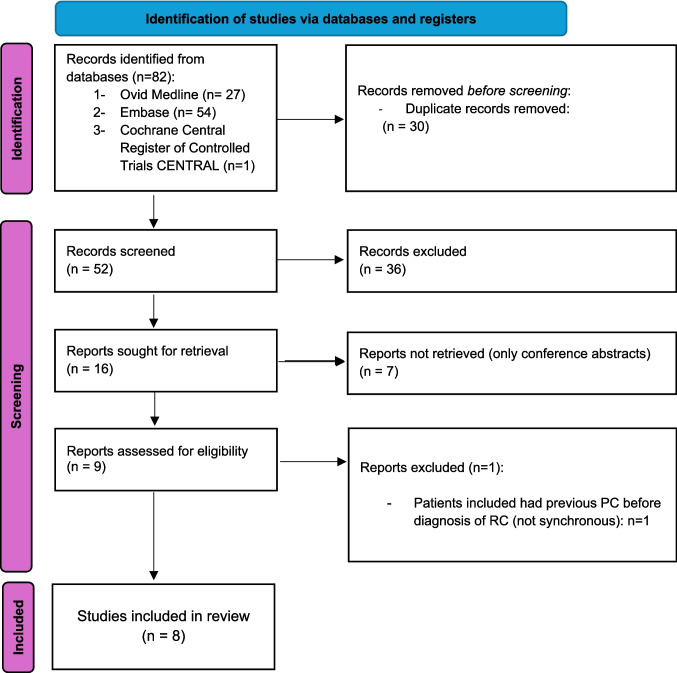
PRISMA diagram

A total of 82 records were identified, of which 52 underwent abstract screening following removal of duplicates. This process resulted in 9 studies being selected for full-text review. Ultimately, eight retrospective case series were included, with only two being multicentre studies [2, 16]. The study selection process is illustrated in the PRISMA diagram (Fig. 1), and a summary of the included studies is provided in Table 1.

**Table 1:** Studies summary

Study, year (country)	Design (Study period)	Risk of bias	No. of patients with synchronous disease	Inclusion criteria	Follow up period	Main focus (Aims)	Treatment strategy	Type of resection, surgical approach and reconstruction	Reported outcomes
Brière et al.,2024 (Canada)	Single centre (2007 to 2021)	Low concerns of bias	16	Synchronous RC and PC, no prior treatment for either cancer, curative intent surgery	Up to 5 years	Evaluate safety/efficacy of synchronous treatments for RC and PC	1) Pelvic CRT + Prostate brachytherapy ± ADT followed by TME (*n* = 10) 2) Pelvic CRT + prostate booster followed by TME (*n* = 4) 3) Rectal brachytherapy + ADT followed by TME (*n* = 1) 4) Pelvic CRT + ADT + TME (n = 1)	**Types of resections:** 1) TME (*n* = 16): AR = 12, APR = 4 **Reconstruction:** 1) Primary anastomosis with no DLI (*n* = 3) 2) primary anastomosis + DLI (*n* = 11) 3) no anastomosis,permanent stoma (n = 2)	Primary: R0, 3-year DFS, OS. Secondary: 30-day severe morbidity
Doussot et al.,2020 (France)	Multi-centre (2008 to 2018)	Moderate concerns of bias	25	Diagnosed within 12 months of each other, curative intent, no distant metastases	Median: 31.2 months	Analyse outcomes of synchronous rectal resection and PC treatments	1) Pelvic CRT + Prostate booster ± ADT followed by TME (*n* = 12) 2) Pelvic radiotherapy followed by TME (*n* = 5) 3) Pelvic CRT + prostate booster followed by pelvic exenteration (*n* = 4) 4) Other (*n*= 4)	**Types of resections:** 1) TME (n = 19): AR = 15, APR = 4, mainly laparoscopic (*n* = 15, 78.9%) 2) Exenteration (en block resection) (*n* = 5) 3) Combined TME + prostatectomy (*n* = 1) **Reconstruction:** 1) Primary anastomosis with no DLI (n = 5) 2) Primary anastomosis + DLI (*n* = 13 2) no anastomosis, permanent stoma (*n* = 7)	R0 resection, mortality, morbidity, RFS, OS
Fukata et al., 2022 (Japan)	Single centre (2018 to 2021)	Major concerns of bias	5	Synchronous RC and PC, suitable for sequential resections, no distant metastases	Median (range): 12 (12–20) months	Report cases treated with simultaneous robotic assisted surgical resection	1) Straight to Surgery: synchronous resection (*n* = 4) 2) neoadjuvant chemotherapy (n = 1)	**Types of resections:** 1) combined TME + prostatectomy, Robotic assisted (n = 5, 100%) **Reconstruction:** 1) Primary anastomosis with no DLI: (*n* = 1) 2) Primary anastomosis + DLI: (*n* = 2) 2) no anastomosis, permanent stoma (n = 2)	Short-term post-op outcomes; bladder function, pad use
Jacobs et al.,2020 (USA)	Multi-centre (1988 to 2017)	Moderate concerns of bias	54	Diagnosed within 6 months, Gleason ≥ 6 for PC, Stage I–III RC, no metastatic disease	Median:43 months	Analyse treatment patterns and outcomes for synchronous RC and PC	1) Pelvic CRT followed by TME (n = 21) 2) Prostatectomy ± RT ± ADT for PC followed by TME (n = 15) 3) Pelvic radiotherapy followed by TME (n = 9) 4) Pelvic CRT followed by prostatectomy and trans-anal local excision (n = 7) 5) Other (n = 2)	**Types of resections:** 1) TME (n = 34): AR = 24, APR = 10, all open (100%) 2) Trans-anal excision and prostatectomy (*n* = 7) 3) Combined TME + prostatectomy (*n* = 1), open 4) Exenteration (enblock resection): (*n* = 1), open 5) Other or no surgery (*n* = 11) **Reconstruction:** 1) no anastomosis, permanent colostomy (n = 19) 2) Primary anastomosis (*n* = 24)	OS, toxicity, leak, local/distant recurrence, mortality
Kavanagh et al., 2012 (Ireland)	Single centre (2000 to 2011)	Moderate concerns of bias	5	Synchronous or metachronous RC and PC, curative intent, suitable for sequential or combined surgery	Mean (SD): 26.4 ± 38.2 months	Describe treatment experience and propose algorithm	1) Pelvic CRT + Prostate booster followed by Exenteration (en-bloc resection):(n = 2) 2) Pelvic CRT + Prostate booster followed by combined prostatectomy and TME (n = 1) 3) Prostatectomy followed by pelvic CRT and TME (n = 1) 3) Pelvic CRT ± ADT followed by Watchful waiting (n = 1)	**Types of resections:** 1) combined TME and prostatectomy (n = 2) 2) Exenteration (enblock resection):(*n* = 2) 3) No surgery (*n* = 1) **Reconstruction:** 1) Primary anastomosis + DLI (n = 1) 2) no anastomosis,permanent stoma (n = 3) 3) Watchful waiting (n = 1)	Post-op morbidity, R0, survival, recurrence
Lavan et at, 2015 (Ireland)	Single centre (not specified)	Moderate concerns of bias	10	Synchronous RC and PC, no prior pelvic radiation, curative intent surgery	Median (Range) 2.2(1.2–6.3) years	Explore feasibility and safety in patients with dual pathology	1) Pelvic CRT + Prostate booster followed by TME (n = 7) 2) Pelvic CRT followed by TME (n = 1) 3) Straight to Surgery (n = 1) 4) Pelvic CRT followed by Watchful waiting (n = 1)	**Types of resections:** 1) TME (n = 8): AR = 7, APR = 1 2) Exenteration (en block resection (n = 1) 3) refused surgery (watchful waiting) (n = 1) **Reconstruction:** 1) no anastomosis, permanent stoma (n = 2) 2) not specified (n = 7)	Early/late toxicity, distant recurrence, R0 resection
Maeda et al., 2021(Japan)	Single centre (2018 to 2020)	Moderate concerns of bias	2	Diagnosed within 12 months, Stage II–III RC, Gleason ≥ 6 PC, no prior pelvic CRT or surgery	No specified – Only short-term post-op follow up	Report clinical impact of robotic-assisted surgery	1) Neoadjuvant chemotherapy followed by Exenteration (n = 1) 2) Pelvic CRT followed by TME (n = 1)	**Types of resections:** 1) Exenteration (enblock resection) (n = 1), Robotic assisted 2) TME (n = 1), Robotic assisted **Reconstruction:** 1) no anastomosis, permanent stoma (n = 1) 2) primary anastomosis + DLI (n = 1)	Operating time, blood loss, negative CRM, post-op complications
Williams et al., 2020 (Australia)	Single centre (2012 to 2016)	Low concerns of bias	7	Synchronous RC and PC, no distant metastases, suitable for robotic-assisted surgery	up to 31 months	Examine outcomes following robotic pelvic exenteration	1) Pelvic CRT followed by TME + prostatectomy (n = 5) 2) ADT followed by TME + prostatectomy (n = 1) 3) Straight to surgery (n = 1)	**Types of resections:** 1) Combined TME and prostatectomy (n = 7), all robotic assisted (100%) **Reconstruction:** 1) no anastomosis,permanent stoma (n = 5) 2) Primary anastomosis + DLI (n = 2)	Operative outcomes, oncological outcomes, time to recurrence

*RC* rectal cancer, *PC* prostate cancer, *CRT* chemoradiotherapy, *ADT* androgen deprivation therapy, *TME* total mesorectal excision, *DLI* diverting loop ileostomy, *DFS* disease free survival, *OS* overall survival, *RFS* recurrence free survival, *CRM* circumferential resection margins

Across the eight included studies, a total of 134 patients were identified with a diagnosis of synchronous RC and PC. However, seven patients from the study by Kavanagh et al. were excluded due to three having non-synchronous RC and PC and four having metastatic disease from an unknown primary origin [17]. Similarly, three patients were excluded from the study by Maeda et al. because they had non-synchronous disease [18]. After these exclusions, 124 patients with synchronous RC and PC who received treatment with curative intent were included in this review.

Across all studies, the median age of patients ranged from 60 to 73 years, and the median body mass index (BMI) ranged from 23 to 28 kg/m2 in five studies [7, 9, 16, 18, 19]. In four studies, the majority of patients were classified as ASA 2 (n = 35, 66%), followed by ASA 1 (n = 9, 16.9%), with the remaining patients categorized as ASA 3 [7, 16, 18, 19]. Additionally, in two studies, 11 patients (26.8%) were reported as active smokers [7, 16] (supplementary Table 4).

Across all studies, T3 was the most common stage of RC, reported in 70 patients (56.5%). This was followed by T2 in 26 patients (21%), T4 in 11 patients (8.9%), and T1 in seven patients (5.6%). A complete pathological response (ypT0) was achieved in two patients (1.6%) in one study [16]. The T stage was unknown in eight patients (6.5%).

Nearly half of the patients, 63 (50.8%), had negative nodal disease (N0) at presentation, while 55 (44.3%) had lymph node involvement (N1 or N2), and six (4.8%) had an unknown lymph node status. Among those with nodal involvement, 13 patients (23.6%) were classified as N2 or higher.

Across three studies, 21 patients (41.1%) had low rectal cancer, 25 patients (49%) had mid-rectal cancer, and 5 patients (9.8%) had upper rectal cancer [7, 16, 20]. In two studies, the median tumour distance from the anal verge ranged from 4 to 7 cm, with one study reporting a median (range) of 4 cm (1–7 cm) and another reporting a median (IQR) of 7 cm (3–9 cm) [16, 19].

Across four studies, PC risk classification was reported as low-risk in 22 patients (20.9%), intermediate-risk in 39 patients (37.1%), and high-risk in 35 patients (33.3%). The risk classification status was unknown in 9 patients (8.5%), and only 3 patients (2.8%) had metastatic disease at diagnosis but remained eligible for curative treatment of RC [2, 7, 16, 20].

In four other studies, PC was locally advanced (T3 or T4) in 36 patients (47.4%) [2, 9, 19, 20]. Notably, the majority of cases were diagnosed incidentally during routine workup investigations for RC [2, 7, 16, 17]. However, in two studies, PSA screening was routinely used for all patients diagnosed with RC, facilitating the early detection of synchronous PC [2, 17]. (supplementary Table 4).

There was significant variation in the management of synchronous RC and PC both within individual studies and across different studies. The most common treatment approach was neoadjuvant pelvic CRT followed by surgical resection with TME alone, combined TME and prostatectomy, or pelvic exenteration. Pelvic CRT followed by surgical resection was used in 36 patients (29%).

The second most common strategy involved delivering a prostate booster dose of RT after neoadjuvant pelvic CRT given for RC, followed by surgical resection, which was employed in 30 patients (24.2%). Additionally, neoadjuvant pelvic CRT for RC was complemented by prostate brachytherapy prior to surgical resection in 10 patients (8%).

A total of 14 patients (11.3%) received preoperative RT for RC alone, without additional chemotherapy or prostate-directed RT, prior to surgical resection. Meanwhile, three patients (2.4%) received only preoperative chemotherapy before undergoing surgical resection.

A small subset of two patients (1.6%) underwent a watchful waiting approach following initial neoadjuvant treatment for both PC and RC with no subsequent surgery, highlighting the individualised nature of treatment decisions for synchronous RC and PC (Table 1).

A total of 111 patients underwent surgical resection, and in 11 patients, it was unknown whether surgical resection was performed.

Among those who had resection, 83 patients (74.7%) underwent TME. Of these, 61 patients (73.4%) had anterior resection, 19 patients (22.9%) underwent extralevator abdominoperineal resection, and three patients (3.6%) had an intersphincteric abdominoperineal resection. Additionally, 12 patients (10.8%) underwent combined TME and prostatectomy, while nine patients (8%) had a pelvic exenteration, where both RC and PC were resected en bloc. Local excision of RC was performed in seven patients (6.3%).

In seven studies, 70 patients (61.4%) underwent open surgery, 27 patients (23.7%) had a laparoscopic approach, 14 patients (12.2%) underwent robotic-assisted surgery, and three patients (2.6%) underwent transanal TME resection [2, 7, 9, 16–19] (Table 1).

Follow-up periods varied across studies, ranging from short-term post-operative assessments to structured long-term surveillance. The median follow-up ranged from 12 to 43 months [7, 9, 16, 20], with one study reporting a mean follow-up of 26.4 ± 38.2 months [17]. One study followed patients every 3–4 months for the first two years, then every 6 months for a total of 5 years or until disease recurrence [7]. One study provided only short-term follow-up [18] (Table 1).

Across multiple studies, post-operative hospital stay varied significantly. The median hospital stay ranged from 6.0 days (IQR: 5.0–8.5) in Brière et al. to 13 days (IQR: 9–18) in Doussot et al. [7, 16]. In Williams et al., where all patients underwent robotic-assisted combined TME and prostatectomy resections, the median stay was 9 days (range: 6–34 days), reflecting variability in recovery times [19]. Kavanagh et al. reported the longest hospital stay, with a mean of 33 days (SD: ± 25.4 days), likely due to the inclusion of patients undergoing either combined TME and prostatectomy or pelvic exenteration [17]. In the remaining studies, hospital stay duration was not specified.

In seven studies [2, 7, 9, 16–19], 63 patients (61.8%) had a primary anastomosis, with 30 patients (77%) receiving a temporary diverting stoma in six studies [7, 9, 16–19]. Meanwhile, 39 patients (38.4%) had a permanent stoma in seven studies [2, 7, 9, 16–19].

Overall, 19 patients (29.2%) had no post-operative complications, while 13 patients (20.0%) experienced Grade 1 complications. Moderate complications (Grade 2) were reported in 10 patients (15.4%). More severe complications occurred in six patients (9.2%) with Grade 3a and eight patients (12.3%) with Grade 3b. Post-operative mortality was observed in two patients that had pelvic exenterations (3.1%) [7, 9, 16, 17, 19].

When comparing studies that exclusively used robotic-assisted surgery [9, 19] to those employing open or laparoscopic approaches [7, 16, 17], patients who underwent robotic-assisted resection had a significantly lower risk of severe complications (Grade 3b–5) compared to non-robotic approaches (8.3% vs. 19.5%, P < 0.0001). However, it is important to note that seven patients (15.2%) in the non-robotic group underwent en-bloc (exenterative) resection, which may have influenced complication rates.

Across studies, colorectal anastomotic leaks occurred in 10.8% of cases (four out of 37 primary anastomoses), with individual study rates ranging from 7.1% to 20% [7, 9, 16, 17, 19]. Vesico-urethral leaks were reported in 40% of cases (two out of five anastomoses), exclusively in one study, Fukata et al. [9] No colorectal or vesico-urethral anastomotic leaks were reported in three studies [17–19].

When comparing colorectal anastomotic leak rates between patients who underwent robotic-assisted resection and those who had non-robotic approaches (open/laparoscopic), the leak rate was lower in the robotic group (7.1%) compared to the non-robotic group (13%). However, this difference was not statistically significant (*P* = 0.051).

In seven studies, R0 resection was achieved in 65 (92.8%) cases [7, 9, 16–20]. There was no significant difference in R0 resection rates between studies that utilised robotic-assisted combined TME and prostatectomy [9, 18, 19] and those that employed other approaches, including open or laparoscopic with different types of surgical resections (TME only, combined TME and prostatectomy or en bloc exenterative resections) [7, 16, 17, 20] (92.8% vs. 94.5%, P = 0.42).

In six studies, seven patients (5.9%) developed locoregional recurrence of RC [2, 7, 9, 16, 19, 20]. In seven studies, 33 patients (27%) experienced RC-related distant metastasis, while clinical or biochemical recurrence of PC was reported in 24 patients (19.6%) [2, 7, 9, 16, 17, 19, 20]. There was no significant difference in the incidence of local recurrence between robotic [9, 19] and non-robotic resections [2, 7, 16, 20] for RC (7.7% vs. 5.7%, P = 0.77).

In two studies, the 3-year DFS for RC ranged from 68.6% to 71.4%, while the 3-year OS ranged from 80.2% to 84.4% [7, 16]. One study reported a median overall survival (OS) of 58 months (range: 36–106 months) [2]. Data on survival outcomes were not specified in other studies.

Across seven studies, RC-related mortality occurred in 24 patients (19.4%), while PC-related mortality was confirmed in only two patients (1.6%). Deaths from other causes were reported in 17 patients (13.7%) [2, 7, 9, 16, 17, 19, 20]. Jacobs et al. had the highest RC-related mortality (33.3%) [2] (Supplementary Table 4).

There was significant variability in the reporting of quality-of-life outcomes across studies. In Brière et al., the median (IQR) I-PSS was 4 (2.3–6.3), indicating mild urinary symptoms (score 1–7). No cases of urethral strictures were reported, and the median CTCAE grading for late side effects was 1, corresponding to mild long-term symptoms [7].

In another study where all patients underwent robotic-assisted resection, three patients (60%) were pad-free at 6 months, and all five patients (100%) had no urinary incontinence (pad-free) at 12 months [9].

Across two studies, 24 patients (37.5%) experienced gastrointestinal side effects of CTCAE Grade 1 or 2, while 10 patients (15.6%) had urinary side effects causing dysfunction of Grade 1 or 2. More severe complications were reported in five patients (7.8%) with Grade 3–4 gastrointestinal side effects and eight patients (12.5%) with Grade 3–4 urinary complications [2, 20].

One study, Fukata et al [9], was rated as having a high risk of bias, while five studies [2, 16–18, 20] were identified as having moderate concern regarding bias. The remaining two studies [7, 19] were categorized as having a low risk of bias. Further details are provided in Table 1 and Supplementary Table 5.

## Discussion

Rectal cancer and prostate cancer are amongst the most common malignancies in males, with their incidence expected to rise due to an aging population [21, 22]. Recent global data show a rising incidence of early-onset rectal cancer in individuals under 50 [23]. While prostate cancer remains more common in older men, there has been a sharp rise among males under 55 over the past two decades, likely due to earlier PSA screening and incidental findings during cancer staging [24]. Additionally, increased life expectancy has contributed to higher detection rates of both cancers, which may help explain the growing clinical relevance of synchronous presentations [25], posing unique challenges in diagnosis and management.

Managing synchronous RC & PC is a complex and challenging clinical task. Moreover, there is a lack of robust evidence to guide clinicians on the optimal treatment strategies [16]. This systematic review was conducted to evaluate the existing literature to aid clinical decision-making in this challenging scenario as well as helping to identify key gaps in the available currently available evidence and guide future research in the topic.

The curative treatment of high-risk PC is challenging when coexisting with RC. High-dose, PC targeted radical RT increases the risk of rectal toxicity and surgical morbidity [26], while curative resection for both cancers requires extensive surgery, increasing risk of complications [27].

In PC, both radical RT and surgical resection provide comparable long-term oncological outcomes [28]. In contrast, RC often requires a combination of neoadjuvant CRT and surgical resection [29]. It is uncertain whether curative treatment of PC improves survival in the presence of synchronous RC, or if treatment should focus on RC to avoid PC-specific interventions that may compromise outcomes of RC specific treatment [7, 20]. These factors underscore the complexity of managing synchronous disease.

The RT dose and field coverage for each cancer differ significantly. PC radiotherapy requires a higher dose (~ 74Gy) with an upper limit at L5/S1, whereas RC radiotherapy requires a lower dose (~ 54Gy) with an upper limit at S1/S2 [16, 30, 31]. This discrepancy can significantly impact outcomes. Higher-dose PC targeted RT may increase surgical complications in RC treatment, while lower-dose RC RT is inadequate for curative-intent PC treatment.

In this study, which included 124 patients with synchronous RC and PC, pelvic CRT (standard chemoradiation for rectal cancer) followed by surgical resection was found to be the most common treatment modality, used in 29% of patients. TME alone was the most frequently performed surgical approach, accounting for 74.4% of resections, whether restorative or non-restorative, highlighting that prostate preserving strategy is a popular treatment option. It has been reported that en-bloc pelvic exenteration carries a significant risk of poor post-operative outcomes [2, 16].

It was noted that postoperative complications (*P* < 0.0001; significant) and colorectal and vesico-ureteric leak rate (*P* = 0.5; non-significant) are lower in patients who underwent robotic assisted resections when compared to other approaches such as open or laparoscopic. However, this result should be interpreted with caution as patients who were offered open resection could have had more complex or advanced disease. This finding should be addressed by RCT comparing open and robotic outcomes in patients with synchronous PC and RC who have comparable staging and surgical procedures.

Robotic surgery is known to offer better platform for pelvic surgery including 3-D vision, 10X magnification and enhanced dexterity of surgical instrument [28]. However, we noted that robotic approach did not offer better outcomes in terms of lower local recurrence rates as there was no significant difference in the incidence of local recurrence between robotic and non-robotic resections for RC (7.7% vs. 5.7%, *P* = 0.77).

There was no statistical difference in R0 resection rate when comparing patients who had combined TME and prostatectomy and those that employed other approaches, where only TME resection was the most adopted type of resection. This indicates that such extensive resection is not routinely needed in all patients and selection of the surgical procedure should be individualised and tailored to the preoperative staging, response to neoadjuvant therapy if used and patient wishes.

There is lack of clear data on DFS and OS in the available literature. The 3-year DFS for RC is reported to be from 68.6% to 71.4% in some studies, while the 3-year OS ranged from 80.2% to 84.4%. However, Data on survival outcomes were not specified in most of the studies. It is recommended that future studies should include clear data on DFS and OS in relationship to various available treatment options as this would guide clinicians in choosing the best treatment strategy to sue for their individual patients.

To our knowledge, this is the first systematic review to evaluate the management of synchronous RC and PC with the largest cohort of patients included in a single study. It provides a comprehensive summary of treatment outcomes and key management considerations, alongside recommendations based on the available evidence. More importantly, this review identifies critical evidence gaps and suggests specific areas for future research (Table 2).

**Table 2:** Summary of recommendations

Topic	Recommendation and summary of current evidence
**CRC screening for patients diagnosed with PC**	- A colonoscopy should be performed for patients diagnosed with PC prior to treatment if not been performed within the last 3 years. This can allow early detection of RC and improve survival (Evidence**:** 1) 28% of patients started treatment for PC (either prostatectomy or radiotherapy) were found to have RC [2]. 2) Number needed to screen to detect synchronous disease = 31 [4])
**Indication and strategy of treatment of PC**	- Priority of management should be for RC rather than PC as RC is a greater contributor to mortality than PC in men with both cancers [2, 7]. = Radical prostatectomy or prostatic booster RT may not be required for high-risk PC and other treatment options can be considered. However, thorough discussion with the patient is required to discuss pros and cons of different options as there is a risk of PC progression after treatment of RC which may require salvage prostatectomy which can be technically challenging due to previous pelvic surgery and RT for RC [2, 7, 16]. - Prostate preserving strategy can improve short and long term post-operative outcomes without compromising survival [7].
**Radiotherapy for synchronous RC and PC**	- Prostate booster RT should not be given if planning combined resections of both PC and RC as there are no added oncological benefit and higher risk of complications [16]. - In prostate preserving surgery for RC, post operative prostate booster RT may decrease anastomotic complications but it is not clear if any long-term benefit compared to pre-operative administration [2]. - Transperineal prostate brachytherapy to supplement EBRT to reach curative dose for PC can decrease post-operative complications for prostate preserving surgery without compromising oncological outcomes [7].
**Chemotherapy**	- Bevacizumab if combined with high dose RT can cause significant long term rectal toxicity if given for LARC [20].
**Surgical resection and anastomosis**	- Pelvic exenteration should be avoided if satisfactory oncological resection can be achieved due to high complication and mortality rates [19]. - Patients should be carefully selected to have bowel anastomosis after high dose RT as there is a high risk of complications and diverting ileostomy should be always considered when performing low anastomosis [2, 9, 16]. - The two-stage Turnbull–Cutait delayed coloanal anastomosis [32] and hand-sewn primary anastomosis are two techniques that may be considered for low anastomosis in combined rectal and prostate resections [7, 16]. - When performed combined resection (TME and prostatectomy), robotically, there is a higher risk of complications (leak, fistula and urosepsis) from vesicourethral anastomosis and this is due to the inability to provide any posterior support of the anastomosis (Rocco stitch) [9]. - Omental flap between both bowel and vesicourethral anastomosis can decrease the risk of development of fistula
**Surgical approach**	- Robotic assisted surgery may improve post-operative outcomes without compromising oncological outcomes [9, 19].
**Suggested research topics**	
- Large retrospective study to assess the prevalence of synchronous RC in patients diagnosed with PC and determine the number needed to screen for early detection - Comparative study evaluating surgical, oncological, and survival outcomes in patients undergoing pelvic CRT followed by TME versus those receiving prostate booster RT with pelvic CRT prior to TME - Comparative study investigating the impact of preoperative vs. postoperative prostate booster RT on surgical outcomes, anastomotic complications, and long-term survival - Comparative study assessing supplementary brachytherapy vs. external beam radiotherapy (EBRT) for curative-intent PC treatment prior to RC surgical resection, focusing on efficacy, toxicity, and postoperative outcomes	

This systematic review has several limitations. Significant heterogeneity among the included studies prevented the conduct of a meta-analysis. The retrospective nature of all included studies poses a risk of reporting bias and inaccuracy in data collection. Additionally, some studies were classified as having moderate to high concerns of bias based on risk assessment criteria. Comparisons within this study are also subject to confounding factors, which may have influenced the results. Despite this, given the lack of high quality RCTs on management of patient with combined RC & PC, this systemic review will give insight to clinicians on the current available treatment strategies for patients presenting with synchronous RC and PC, and their outcomes.

In conclusion, this review evaluates treatment outcomes for patients with synchronous RC and PC and provides evidence-based recommendations. There is no consistent approach in management of synchronous RC and PC. High dose radiotherapy targeting PC may not offer additional survival benefit while increasing the risk of post-operative complications. Robotic assisted resections for both RC and PC may decrease risk of major post-operative complications without compromising oncological outcomes.

## Supplementary Information

Below is the link to the electronic supplementary material.

Supplementary file1 (DOCX 40 KB)

## Data Availability

No datasets were generated or analysed during the current study.
