# Supplementary material for: Outcomes of the management of synchronous rectal and prostate cancer: a systematic review
Source: Int J Colorectal Dis. 2025 Sep 11;40(1):195. doi: 10.1007/s00384-025-04992-w (PMC12423125; doi:10.1007/s00384-025-04992-w)
Supplement: Supplementary file 1 — Supplementary file1 (DOCX 40 KB) [file 384_2025_4992_MOESM1_ESM.docx]

**Supplementary Material**

**Contents:**

- Supplementary table 1: PICO framework
- Supplementary table 2: Search strategy (Ovid MEDLINE)
- Supplementary Table 3: Definitions
- Supplementary table 4: Demographics, disease specific data and outcomes:
- Supplementary table 5: risk of bias for case series

**Supplementary table 1: PICO framework**

| **Population(s)** | Patients with synchronous rectal and prostate cancer |
| --- | --- |
| **Intervention(s) & Comparators** | Pelvic exenteration, combined resection of rectal and prostate cancer, TME only and different chemoradiotherapy regimens. |
| **Outcomes** | Post-operative complications and hospital stay, oncological and survival outcomes and quality of life |

**Supplementary table 2: Search strategy (Ovid MEDLINE)**

| **Database:** | **Ovid MEDLINE(R) ALL <1946 to February 18, 2025, >** | **Results per line:** | **Number of results: 27** |
| --- | --- | --- | --- |
| **Date:** | **19/02/2025** |  |  |
| 1 | Synchronous.ti,ab,kw,kf. | 46387 |  |
| 2 | Rectal Neoplasms/ | 50075 |  |
| 3 | ((rect* adj2 cancer*) or (rect* adj2 neoplasm*) or (rect* adj2 tumo?r)).ti,ab,kw,kf. | 38073 |  |
| 4 | 2 or 3 | 61731 |  |
| 5 | Prostatic Neoplasms/ | 148487 |  |
| 6 | ((Prostat* adj2 cancer*) or (prostat* adj2 neoplasm*) or (prostat* adj2 tumo?r)).ti,ab,kw,kf. | 163916 |  |
| 7 | 5 or 6 | 198838 |  |
| 8 | 4 and 7 | 1211 |  |
| 9 | Neoadjuvant Therapy/ | 30964 |  |
| 10 | Brachytherapy/ | 22297 |  |
| 11 | exp Chemoradiotherapy/ | 21067 |  |
| 12 | Prostatectomy/ | 33351 |  |
| 13 | Radiotherapy/ | 44520 |  |
| 14 | Pelvic Exenteration/ | 1893 |  |
| 15 | (Brachytherap* or Total mesorectal excision* or "TME" or Neoadjuvant Therap* or chemoradi* or prostatectom* or radiotherap* or synchronous radical treatment* or synchronous manage* or (synchronous adj2 treat*) or curative intent management* or (simultaneous adj2 manage*) or (simultaneous adj2 treat*) or Pelvic exenteration* or (surger* adj2 remov*) or anterior resection* or prostatectom*).ti,ab,kw,kf. | 349749 |  |
| 16 | 9 or 10 or 11 or 12 or 13 or 14 or 15 | 402700 |  |
| 17 | 1 and 8 | 42 |  |
| 18 | 16 and 17 | 27 |  |
| 19 | limit 18 to english language | 27 |  |

**Supplementary Table 3: Definitions**

| **Term** | **Definition** |
| --- | --- |
| **Pelvic CRT** | Standard CRT regimen for RC as specified by each study with RT dose not exceeding 54 Gy |
| **Prostate booster** | Additional RT added to total RT dose to meet requirement for curative RT for PC (>64Gy) or as defined by individual study to be the curative dose for PC |
| **Combined TME and prostatectomy** | A synchronous but separate resection of both RC and PC during the same procedure where both specimens retrieved separately. |
| **En-bloc resection (exenteration)** | Both rectal and prostate specimens retrieved intact as one inseparable specimen |
| **Locations for RC** | Low: <5cm from anal verge; Intermediate: from 5 to 10 cm from anal verge; high: >10 cm from anal verge |
| **Risk classification for PC** | PC classified into low, intermediate and high risk of recurrence according to D'amico risk classification system ^1^ or as defined by each individual study. |

CRT: chemoradiotherapy; RT: radiotherapy; TME: total mesorectal excision; RC: rectal cancer; PC: prostate cancer

**Supplementary table 4: Demographics, disease specific data and outcomes:**

| **Study ID** | **Age (Range)** | **Disease specific details** | | | | **Outcomes** | | | | | | |
| --- | --- | --- | --- | --- | --- | --- | --- | --- | --- | --- | --- | --- |
|  |  | **T stage (RC)** | **N Stage (RC)** | **Risk assessment and staging for PC** | **Metastasis at presentation** | **30-Day Post-Op Complications** | **Anastomotic Leak** | **Permanent stoma formation** | **Length of Hospital Stay**  **Median (IQR)** | **R0 resection and Recurrence Rates** | **Survival Outcomes**  **Median (95%CI) %** | **Mortality** |
| **Brière et al** | 72 (68-75) | cT2=4 (25%),  cT3=12 (75%) | cN0=9 (56.3%),  cN1=7 (43.7%) | Low risk: n=3 (18.8%)  Intermediate risk: (n= 8 (50%)  High-risk: n=5 (31.3%) | RC: None  PC: 1 patient | None: 7 (43.8%), Grade 1: 1 (6.3%), Grade 2: 2 (12.5%), Grade 3b: 6 (37.6%) | 1 out from 14 primary anastomosis (7.1%) | 2 (12.5%) | 6.0 (5.0-8.5) days | **R0**: 15 (93.8%)  **RC related**: a) local: 2(12.5%), b) distant: 4(25%)  **PC related:** 0% | **3-year DFS for RC**: 71.4 (40.2-88.3)  **3-year OS:** 84.4 (50.4-95.9) | **RC-related**: 2 (12.5%)  **PC-related**: 0  **Other cause**: 3 (18.8%) |
| **Doussot et al** | 71 (67-75) | ypT0 : n=2 (8%)  ypT2: n=10 (40%)  ypT3: n= 11 (44%)  ypT4: n=2 (8%) | cN0: n=17 (68%)  cN1: n=7 (28%)  cN2: n=1 (4%) | Low risk: n=9 (36%)  Intermediate risk: n=10 (40%)  High risk: n=6 (24%) | RC: None  PC: None | None: 9 (36%)  Grade 1: 5 (20%)  Grade 2: 6 (24%)  Grade 3a: 3 (12%) Grade 4 or 5: 2 (8%) | 2 of 18 primary anastomosis (11%) | 10 (40%) | 13 (9-18) days | **R0**: 24 (96%)  **RC related:** a) local: 0  b) distant: 3(12%)  **PC related**: 0% | **3-year DFS for RC**: 68.6 (42.3-84.8)  **3-year OS:** 80.2 (58.8-92.2) | **RC-related**: 0  **PC-related**: 0  **Other cause:** 2 (8%) |
| **Fukata et al** | 72 (63-74) | cT3=3 (60%), T2=2 (40%) | cN0=4 (80%), cN3=1 (20%) | cT3: n=2 (40%)  cT2: n=3 (60%)  cN0=5 (100%) | RC: None, PC: ns | None: 2 (40%)  Grade 3a: 2 (40%)  Grade 3b: 1 (20%) Grade 4 or 5: 0 (0%) | 1 (20%) – colorectal leak  2 (40%) Vesico-uretheral leak | ns | ns | **R0**: 5 (100%)  **RC related**: a) local: 0 b) distant: 0  **PC related:** 1 (20%) | ns | **RC-related:** 1 (20%)  **PC-related:** 0 (0%)  **Other cause:** 0(0%) |
| **Jacobs et al** | 67 (62-72) | cT1: n=6 (11.1%)  cT2: n=9 (16.7%)  cT3: n=34 (63%)  cT4: n=2 (3.7%)  Unknown: n=3 (5.6%) | cN0: n=27 (50%), cN1: n=19 (35.2%), cN2: n=5 (9.3%), Unknown: n=3 (5.6%) | Low risk: n= 8 (14.8%)  Favourable intermediate risk: n= 8 (14.8%)  Unfavourable intermediate risk: n=.10 (18.5%)  High risk: n= 19 (35.2%) | RC: None, PC: 2 patients | ns | ns | ns | ns | **R0**: ns  **RC related**: a) local: 4(7.4%). b) distant: 20 (37%)  **PC related**: 19 (35.2%) | **Median (Range) OS**: 58 (36-106) months | **RC-related:** 18 (33.3%)  **PC-related:** 2 (3.7%)  **Other cause:** 11 (20.4%) |
| **Kavanagh et al** | 67.8±10.3 | T3=3 (60%), T4=1 (20%), Unknown=1 (20%) | cN0: n=2 (40%),  cN1: n=2 (40%), Unknown: n=1 (20%) | ns | RC: None, PC: None | None: 1  Grade 1: 3  Grade 3a: 1 (Includes intra-abdominal collection)  Grade 4 or 5: 0 | 0 | 0 | Mean (SD) 33±25.4 days | **R0**: 3 (75%)  **RC related**: a) local: ns b) distant: 1(20%)  **PC related**: 1 (20%) | ns | **RC-related**: 1 (20%)  **PC-related:** 0 (0%)  **Other cause:** 1 (20%) |
| **Lavan et al** | Median 68 (47-79) | T2: n=1 (10%),  T3: n=7 (70%),  T4: n=2 (20%) | cN0: n=5 (50%),  cN1: n =4 (40%), Unknown:  n =1 (10%) | Low risk: n = 2(20%)  Intermediate risk: n= 3 (30%)  High risk: n= 1 (10%)  Very high: n= 4(40%) | RC: None  PC: ns | ns | ns | 2 (20%) | ns | **R0**: 10 (100%)  **RC related**: a) local: 0. b) distant: 3(30%)  **PC related**: 2 (20%) | ns | **RC-related:** 1 (10%)  **PC-related:** 0 (0%)  **Other cause:** 0 (0%) |
| **Maeda et al** | 73 (68.5-78.5) | T4: n=1 (50%)  Unknown: n=1(50%) | ns | ns | ns | ns | 0 | ns | ns | **R0**: 2 (100%)  **RC related**: a) local: ns b) distant: ns  **PC related:** ns | ns | ns |
| **Williams et al** | 60 (46-79) | T1: n=1 (14.3%)  T2: n=1 (14.3%)  T4: n=3 (42.9%)  Unknown: n=2(28.5%) | N0: n= 4 (57%)  N1: n= 2 (28.5%)  N2: n= 1 (14.3%) | T2c: n = 1 (14.3%)  T3a: n= 2 (28.5%)  T4: n = 2 (28.5%)  Unknown: n=2 (28.5%) | RC: None  PC: ns | None: 0  Grade 1: 4 (Includes 4 cases of ileus, 1 case of UTI)  Grade 2: 2 (Includes perineal wound dehiscence, ureteric stricture requiring stent)  Grade 3b: 1 (Includes vesicourethral anastomotic leak and sepsis)  Grade 4 or 5: 0 | 0 | 5 (71.4%) | 9 (6–34) days | **R0**: 6 (85.7%)  **RC related**: a) local: 1(14.2%). b) distant: 2(28.5%)  **PC related**:1(14.2%) | ns | **RC-related:** 1 (14.3%)  **PC-related:** 0 (0%)  **Other cause**: 0 (0%) |

ns: not specified; RC: rectal cancer; PC: prostate cancer; DFS: disease free survival; OS: overall survival; SD: standard deviation

**Supplementary table 5: risk of bias for case series**

|  | **Brière et al** | **Doussot et al** | **Fukata et al** | **Jacobs et al** | **Kavanagh et al** | **Lavan et al** | **Maeda et al** | **Williams et al** |
| --- | --- | --- | --- | --- | --- | --- | --- | --- |
| **Were there clear criteria for inclusion in the case series?** | yes | yes | yes | yes | yes | yes | yes | yes |
| **Was the condition measured in a standard, reliable way for all participants included in the case series?** | yes | yes | yes | unclear | yes | yes | yes | yes |
| **Were valid methods used for identification of the condition for all participants included in the case series?** | yes | yes | yes | yes | yes | yes | yes | yes |
| **Did the case series have consecutive inclusion of participants?** | yes | yes | yes | yes | yes | unclear | yes | yes |
| **Did the case series have complete inclusion of participants?** | yes | yes | yes | yes | yes | yes | yes | yes |
| **Was there clear reporting of the demographics of the participants in the study?** | yes | yes | yes | yes | no | yes | yes | yes |
| **Was there clear reporting of clinical information of the participants?** | yes | yes | no | yes | yes | yes | yes | yes |
| **Were the outcomes or follow-up results of cases clearly reported?** | yes | unclear | unclear | yes | yes | yes | unclear | yes |
| **Was there clear reporting of the presenting site(s)/clinic(s) demographic information?** | yes | yes | unclear | yes | yes | yes | yes | yes |
| **Was statistical analysis appropriate?** | yes | yes | NA | yes | yes | yes | yes | yes |

**References:**

1. Hernandez DJ, Nielsen ME, Han M, Partin AW. Contemporary evaluation of the D’amico risk classification of prostate cancer. *Urology*. 2007;70(5):931-935. doi:10.1016/j.urology.2007.08.055
